# Enhancing the efficiency of planar heterojunction perovskite solar cells via interfacial engineering with 3-aminopropyl trimethoxy silane hydrolysate

**DOI:** 10.1098/rsos.170980

**Published:** 2017-12-20

**Authors:** Ya-Qiong Wang, Shou-Bin Xu, Jian-Guo Deng, Li-Zhen Gao

**Affiliations:** 1 College of Environmental Science and Engineering, Taiyuan University of Technology, Taiyuan, Shanxi 030024, People's Republic of China; 2 China Academy of Engineering Physics, Institute of Chemical Materials, Mianyang, Sichuan 621900, People's Republic of China

**Keywords:** 3-aminopropyl trimethoxy silane, interfacial engineering, perovskite solar cells

## Abstract

The interfacial compatibility between compact TiO_2_ and perovskite layers is critical for the performance of planar heterojunction perovskite solar cells (PSCs). A compact TiO_2_ film employed as an electron-transport layer (ETL) was modified using 3-aminopropyl trimethoxy silane (APMS) hydrolysate. The power conversion efficiency (PCE) of PSCs composed of an APMS-hydrolysate-modified TiO_2_ layer increased from 13.45 to 15.79%, which was associated with a significant enhancement in the fill factor (FF) from 62.23 to 68.04%. The results indicate that APMS hydrolysate can enhance the wettability of γ-butyrolactone (GBL) on the TiO_2_ surface, form a perfect CH_3_NH_3_PbI_3_ film, and increase the recombination resistance at the interface. This work demonstrates a simple but efficient method to improve the TiO_2_/perovskite interface that can be greatly beneficial for developing high-performance PSCs.

## 1. Introduction

Since the first report on perovskite solar cells (PSCs) in 2009 by Miyasaka *et al*., the power conversion efficiencies (PCEs) of PSCs have rapidly increased from approximately 3.8 to over 20% [1,2]. Two dominant device structures, known as mesostructured and planar heterojunction (PHJ) cells, have been employed to fabricate PSCs [3]. The most efficient PSCs employ mesoscopic metal oxides, such as TiO_2_ or Al_2_O_3_, as a scaffold. The mesoporous layer plays a critical role by facilitating the formation of a homogeneous perovskite film and reducing the contact resistance. Al_2_O_3_ is unable to assist in electron extraction due to its large bandgap, which suggests that perovskite itself transports electrons [4]. Hence, PHJ structures have attracted increasing interest due to their potential to simplify the fabrication process by eliminating the need for the high-temperature-sintered mesoporous layer [5]. In a heterojunction structure, with no mesoscopic scaffold, the interfacial connection between compact TiO_2_ and perovskite plays an important role in improving the cell performance. Therefore, other methods must be employed to provide a smooth, continuous perovskite film and to suppress electron–hole recombination [6]. Interfacial engineering is considered an effective method for achieving high device performance for PHJ PSCs. Generally, engineering the interface between a compact TiO_2_ layer and a perovskite layer provides several advantages, such as easier charge transfer from the perovskite to the electron-transport layer (ETL), less interfacial charge recombination, improved perovskite grains and a passivated TiO_2_ surface [7].

Different methods for treating the surface of TiO_2_ have been developed to improve the compatibility of the TiO_2_/CH_3_NH_3_PbI_3_ interface. Qin *et al*. [8] evaporated Cs_2_CO_3_ on a compact TiO_2_ layer, and the corresponding PCE was increased from 8.0 to 11.1%. Shih *et al*. and Y. Ogomi *et al*. applied amino acids to modify the mesoporous TiO_2_/CH_3_NH_3_PbI_3_ heterojunction interface, and the PCE of the resulting PSCs increased from 8.35 to 12.02% and 8 to 10%, respectively [9,10]. Zuo *et al*. [7] used 3-aminopropanoic acid to modify the compact TiO_2_/CH_3_NH_3_PbI_3_ heterojunction, and the PCE increased from 11.96 to 15.67%. However, the area of their solar cell was 5.2 mm^2^, which is substantially different from the typical cell area of 0.1 cm^2^. Other treatments have been employed to modify the surface of TiO_2_ layer, such as TiCl_4_ and UV(O_3_) treatment [11]. Therefore, it is still necessary to develop a simple, fast and efficient method for fabricating highly efficient perovskite solar cells with highly device stability and reproducibility.

Amino silanes are widely applied to improve the quality of active layers. Mallakpour *et al*. [12] used a 3-aminopropyltriethoxy silane coupling agent to modify the OH-rich surface of α-MnO_2_ following hydrolysis. Krishnaiah *et al*. [13] grafted hydrolysed 3-aminopropyltriethoxysilane onto Hal nanotubes in a solution of water and ethanol. This type of surface modification method is very simple, which avoids the use of heat or a complex atmosphere. Owing to the enhanced adhesion between the compact TiO_2_ and perovskite active layers, high-quality CH_3_NH_3_I_3_ films can be fabricated using a one-step spin-coating method [11]. To the best of our knowledge, the use of 3-aminopropyl trimethoxy silane (APMS) in modifying the TiO_2_ layer to improve the PCE of PSCs has not been reported previously.

In this work, we propose to modify the TiO_2_/CH_3_NH_3_PbI_3_ interface by introducing APMS, a type of amino silane, as a coupling agent. This approach enhanced the PCE of the PHJ PSCs from 13.45 to 15.79%, representing a 17.4% enhancement.

## 2. Material and methods

### 2.1. Material and reagents

Fluorine-doped tin oxide glass (FTO) was obtained from YingKou OPV Tech New Energy Co. Ltd. Acetone, isopropanol, *n*-butyl alcohol and APMS were obtained from Aladdin. Bis(pentane-2,4-dionato-O,O′)bis(propan-2-olato)titanium, PbI_2_, γ-butyrolactone (GBL), *tert*-butylpyridine (tBP) and lithium bis(trifluoromethylsulfonyl)imide (Li-TFSI) were purchased from Sigma-Aldrich. CH_3_NH_3_I and spiro-MeOTAD were purchased from Xi'an Polymer Light Technology Corp.

### 2.2. Preparation of the compact TiO_2_ layer

The transparent conducting FTO substrates were cleaned sequentially with 20 min of ultrasonication in detergent, deionized water, acetone and isopropanol, followed by drying in a N_2_ stream. Subsequently, the FTO substrate was placed in UV-irradiation instrument to remove residual organic matter. A compact TiO_2_ precursor solution was synthesized using a sol–gel method, wherein 0.5 ml of bis(pentane-2,4-dionato-O,O′)bis(propan-2-olato)titanium was added to 2 ml of *n*-butyl alcohol and mixed for 5 h at room temperature. The sol was spin-coated onto the clean substrates at 5000 r.p.m. for 30 s, followed by annealing. After drying on a heating platform at 100°C in air, the TiO_2_-coated samples were calcined in a muffle furnace by slowly increasing the temperature (5°C min^−1^) to 500°C, maintaining this temperature for 30 min, and then naturally cooling the product to room temperature.

### 2.3. Modification of the TiO_2_ layer by 3-aminopropyl trimethoxy silane hydrolysate

APMS was hydrolysed in an 80 : 20 w/w mixture of ethanol and deionized water at a concentration of 10% w/w and with acetic acid added at 5% w/w relative to the solvent. The FTO substrate with the compact TiO_2_ layer was immersed into the hydrolysate, and the grafting reaction was sustained for 2 h [14]. After that, the TiO_2_ layer with APMS hydrolysate was washed with water and then immersed into 0.5 mol l^−1^ HI solution for 2 h. Subsequently, the treated compact TiO_2_ layer was washed by deionized water and dried in a N_2_ stream.

### 2.4. Fabrication of PSCs

MAI and PbI_2_ were added into a mixture of GBL and DMSO (7 : 3 v/v) at 60°C for 12 h. The precursor solution was coated onto the TiO_2_ layer with and without APMS modification at 5000 r.p.m. for 55 s. During the spin-coating process, the substrate was treated by chlorobenzene drop-casting [15]. The substrate was then dried on a hot plate at 100°C for 10 min. A spiro-MeOTAD solution was prepared by dissolving 72.3 mg of spiro-MeOTAD in 1.0 ml of chlorobenzene, into which 28.8 µl of tBP and 17.5 µl of a Li-TFSI solution (520 mg Li-TFSI in 1 ml acetonitrile, Sigma-Aldrich, 99.8%) were added. The spiro-MeOTAD solution was spin-coated onto the perovskite films at 5000 r.p.m. for 30 s. Finally, an Au electrode with a thickness of 80 nm was thermally evaporated onto the spiro-MeOTAD-coated substrates.

### 2.5. Characterization

The surface of bare compact TiO_2_ (c-TiO_2_) and APMS-hydrolysate-treated c-TiO_2_ was investigated using X-ray photoelectron spectroscopy (XPS, ESCALAB-210) with Al K*α* radiation (1486.6 eV). The current density (J)–voltage (V) characteristics were measured with a computer-controlled Keithley 2400 under AM 1.5 illumination (100 mw cm^−2^) from a Newport solar simulator. The incident photon-to-electron conversion efficiency (IPCE) was measured by a QEXL Solar Cell Spectral Response instrument. Contact angles were measured by a KRUSS DSA30. The morphology of c-TiO_2_/perovskite and c-TiO_2_/APMS-hydrolysate/perovskite was characterized using field emission scanning electron microscopy (FE-SEM, Hitachi S-4800). X-ray diffraction (XRD) patterns of the CH_3_NH_3_PbI_3_ films were recorded on a HaoYuan DX-700 diffractometer. Capacitance–voltage (C–V) measurements and electrochemical impedance spectroscopy (EIS) were performed on an Electrochemical Workstation (VMP3, Bio-Logic, France).

## 3. Results and discussion

**Figure 1.**
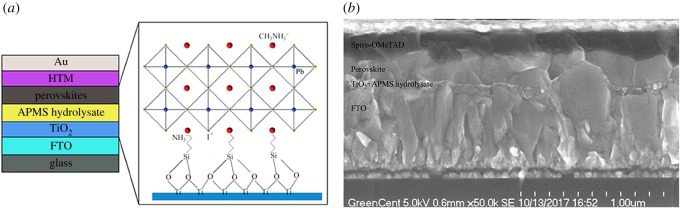
(*a*) Schematic of the perovskite solar cell with APMS hydrolysate inserted between the perovskite and compact TiO_2_ layer. (*b*) Cross-sectional SEM image of the fabricated cell.

The n-i-p planar PSC structure with APMS hydrolysate between TiO_2_ and CH_3_NH_3_PbI_3_ layer is shown in figure 1*a*. Cross-sectional SEM image exhibits each functional layer in figure 1*b*. (HO)_3_-Si-R-NH_3_I was expected to grow on the surface of the compact TiO_2_ layer, in which NH_3_^+^ and I^−^ moieties were incorporated into the surface of the CH_3_NH_3_PbI_3_ layer, thus anchoring it to the perovskite layer [7,10].

**Figure 2:**
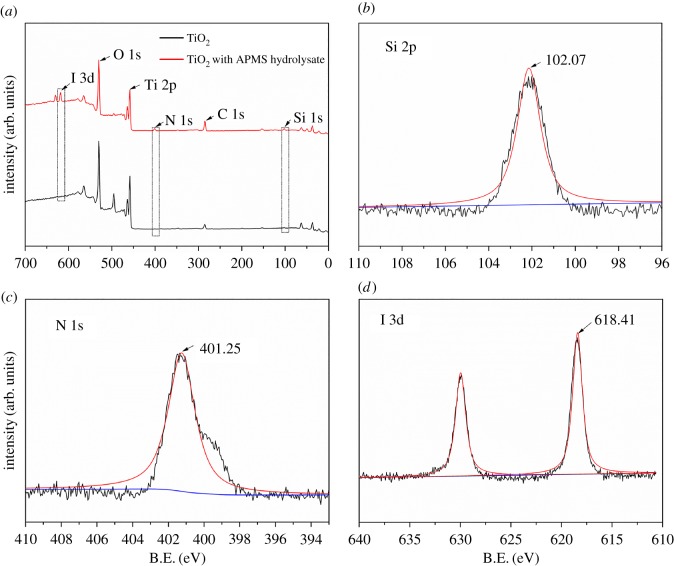
XPS spectrum of the films before and after modification with APMS hydrolysate. (*a*) XPS wide-scan survey, (*b*) Si 2p, (*c*) N 1s and (*d*) I 3d.

As seen in figure 1*b* and electronic supplementary material, figure S1, no obvious modified layer can be observed. The APMS hydrolysate layer was considered as self-assembly monolayer or extremely thin layer, covering the TiO_2_ surface [16]. To investigate the hydrolytic degree of APMS and the grafting onto the surface of the compact TiO_2_ layer, X-ray photoelectron spectroscopy (XPS) was used to analyse the TiO_2_ film before and after surface modification [17]. The XPS wide-scan survey spectra are shown in figure 2*a*. All of the peaks were calibrated using the C 1s peak (284.8 eV) as a reference [18]. Peaks of Si 2p, N 1s and I 3d can be clearly observed in the XPS spectra of TiO_2_ before and after modification with APMS (indicated by rectangles in figure 2*a*). A detailed analysis of these XPS spectra provides clear evidence that the films were chemically modified, which was confirmed by the Si 2p, N 1s and I 3d spectra from fitted curves obtained using XPSPEAK software. As illustrated in figure 2*b*, the Si 2p peak was located at 102.07 eV following modification, while N 1s was at 401.25 eV (figure 2*c*), and the peak for I was located at 618.41 eV (figure 2*d*). Thus, the APMS hydrolysis product was clearly grafted onto the hydroxyl-rich TiO_2_ layer.

**Figure 3.**
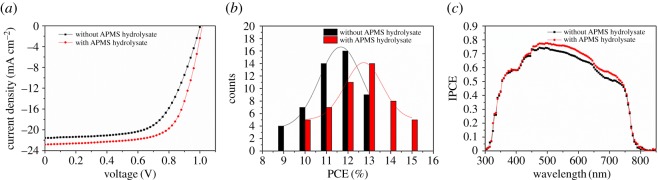
(*a*) J–V curves of the best perovskite solar cells with bare and APMS-hydrolysate-treated TiO_2_ under 100 ms of delay time. (*b*) Histograms of the PCEs of 100 PSCs based on bare and APMS-hydrolysate-treated TiO_2_. (*c*) IPCE spectra of the devices with and without APMS hydrolysate modification.

The current density (J)–voltage (V) characteristics of the PSCs both without and with APMS hydrolysate modification, measured under reverse scan are shown in figure 3*a*. Table 1 shows the performance parameters of the two types of PSCs. The PCE of the solar cells increased from 13.45% when fabricated with a bare compact TiO_2_ layer to 15.79% with the TiO_2_ layer modified by APMS hydrolysate. Moreover, the *J*_sc_ increased from 21.56 to 22.84 mA cm^−2^, and the FF increased from 62.23 to 68.04%. The photovoltaic properties of fabricated devices were examined under reverse and forward scan, as seen in electronic supplementary material, figure S2(*a*,*b*). Both devices exhibit hysteresis. The best PCE under reverse scans is 2.2–2.8% higher than those under forward scans. According to the forward and reverse statistic, it is evident that the hysteresis of the devices was reduced after APMS hydrolysate modification. The PCE of reverse scan was enhanced from 13.45 to 15.79%, it was a 17.4% enhancement, while the PCE of forward scan was enhanced from 10.65 to 13.56%, it is a 27.3% enhancement. Such a different enhancement in PCE was mainly due to the changing rate in FF gap between forward scan and reverse scan was decreased by inserting APMS hydrolysate. Fifty cells for each type of PSC were constructed, and a histogram of their PCEs is shown in figure 3*b*, which clearly demonstrates that the fitted curve shifted right to a higher efficiency. This shift indicates that the average efficiency of the PSCs with modified compact TiO_2_ layers is higher than that of the cells with unmodified layers. Using another analysis method, the PCEs of PSCs modified with APMS hydrolysate exhibit an efficiency range of 10–15%, which is higher than that of unmodified cells, 8–13%. In order to further demonstrate the statistics, a PC_60_BM layer was introduced as an interface layer as reported [19–21]. As a control group, the only difference in the experiment was to replace APMS hydrolysate with PC_60_BM. PCBM solution was spin-coated onto the clean substrates at 6000 r.p.m. for 40 s. The cross-sectional SEM image is shown in electronic supplementary material, figure S1*b*. The best device obtained a PCE of 14.82% with a *V*_oc_ of 1.0 V, a FF of 64.73% and a *J*_sc_ of 22.75 mA cm^−2^.

**Table 1.** Performance data of the perovskite solar cells without and with modification.

sample			*J*_sc_ (mA cm^−2^)	*V*_oc_ (V)	FF	PCE (%)
bare TiO_2_	averaged	forward	20.40 ± 1.25	0.90 ± 0.08	48.40 ± 2.07	9.08 ± 1.37
		reverse	20.36 ± 1.20	0.95 ± 0.05	60.13 ± 2.10	11.89 ± 1.56
	best	forward	21.65	0.98	50.47	10.65
		reverse	21.56	1.00	62.23	13.45
APMS-hydrolysate/TiO_2_	averaged	forward	21.28 ± 1.67	0.92 ± 0.07	56.72 ± 2.99	12.11 ± 1.45
		reverse	21.14 ± 1.70	0.98 ± 0.04	64.81 ± 3.23	14.20 ± 1.59
	best	forward	22.95	0.99	59.71	13.56
		reverse	22.84	1.02	68.04	15.79

Figure 3*c* presents the incident photon-to-electron conversion efficiency (IPCE) spectra of the PSCs. The curves of all of the PSCs display a wide photoresponse from 350 to 800 nm, which is consistent with the absorption spectrum of CH_3_NH_3_PbI_3_. Photocurrent generation was initiated at 1.55 eV, which is in good agreement with the bandgap of CH_3_NH_3_PbI_3_ [22]. The integrated photocurrents estimated from the IPCE curve were 21.1 and 20.0 mA cm^−2^ for the two devices with and without APMS hydrolysate, respectively, which agree well with the values obtained from the J–V measurements. Moreover, the device with APMS hydrolysate obviously demonstrates higher IPCE values, especially in the range of 450–750 nm, as evidenced by the higher IPCE value exhibited at 500 nm (77.6%) related to its bare compact-TiO_2_ ETL-based counterpart (74.0%). This higher IPCE may benefit from more efficient electron collection and less charge recombination due to the insertion of APMS hydrolysate, thereby increasing the *J*_sc_.

**Figure 4.**
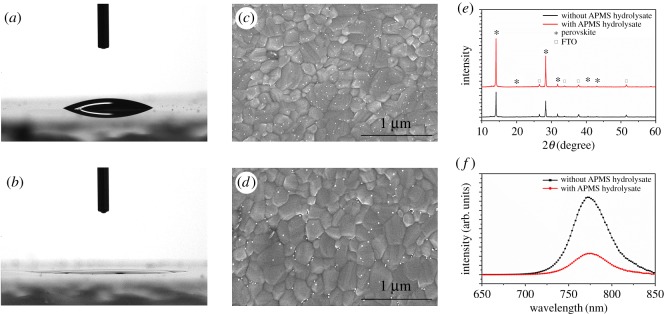
(*a*) GBL droplet exhibiting a contact angle of 23.83° and (*b*) spreading on the TiO_2_ surface without and with APMS hydrolysate treatment, respectively. Top-view SEM images of the perovskite films (*c*) without and (*d*) with APMS hydrolysate treatment. (*e*) XRD patterns of perovskite films on bare TiO_2_ (black line) and modified TiO_2_ (red line) substrates. (*f*) Steady-state photoluminescence (PL) spectra of the perovskite films on bare c-TiO_2_ and on APMS-hydrolysate-modified c-TiO_2_.

The contact angles of droplets of GBL on the modified and unmodified TiO_2_ films were measured to investigate the mechanism through which APMS hydrolysate improves the efficiency of the PSCs [23]. As shown in figure 4*a,b*, the contact angle of GBL on the untreated TiO_2_ film is 23.83°, while the GBL spreads out on the APMS-hydrolysate-modified TiO_2_ film. These results suggest that the GBL wettability on TiO_2_ was enhanced.

SEM images of the perovskite on both the bare and modified TiO_2_ layers are shown in figure 4*c,d*. The perovskite crystals on the modified TiO_2_ layer are more uniform than those on the bare TiO_2_ layer, and the crystals grown directly on the bare TiO_2_ surface exhibit a slightly smaller grain size than those grown on the modified surface. The morphological evolution of the perovskite film with APMS hydrolysate could be attributed to the improved miscibility of the substrate with the perovskite, wherein the amino group is expected to become ammonium and incorporate into the crystalline structure of the perovskite, as shown in figure 1. Larger grain sizes result in fewer grain boundaries for the photogenerated charges to traverse, thereby decreasing charge losses due to recombination at grain boundaries [24].

Figure 4*e* illustrates the X-ray diffraction (XRD) patterns of the CH_3_NH_3_PbI_3_ films grown on TiO_2_ both with and without APMS hydrolysate modification. The peaks at 14.1°, 28.4° and 42.1° can be attributed to the (110), (220) and (330) faces of the CH_3_NH_3_PbI_3_ crystalline structure, respectively [7]. The characteristic peaks appear at the same angles, indicating the pure perovskite phase on both surfaces without a change in the crystal orientation [25]. In addition, the diffraction peaks at 14.1° and 28.4° were significantly enhanced by APMS hydrolysate modification, indicating an improvement in the crystallinity of the CH_3_NH_3_PbI_3_ film.

The steady-state PL spectra of the CH_3_NH_3_PbI_3_ films are shown in figure 4*f* to illustrate the charge transport and dynamics of the corresponding devices fabricated on the c-TiO_2_ layer without and with APMS hydrolysate treatment. The samples were excited at 440 nm, and both perovskite films exhibited an emissive band with a maximum at approximately 770 nm and a broad emission band ranging from 720 to 850 nm. With the introduction of APMS hydrolysate, a significant fluorescence quenching of perovskite is exhibited, which indicates enhanced electron transport from the perovskite to the APMS-hydrolysate-treated c-TiO_2_ layer. This phenomenon demonstrates that the introduction of APMS hydrolysate could facilitate charge transfer between the perovskite and TiO_2_ layer. Owing to the shorter diffusion length of electrons than that of holes in perovskite materials, efficient electron transfer and extraction balance the electron and hole transport in PSC devices, resulting in a significant enhancement of the FF [17].

**Figure 5.**
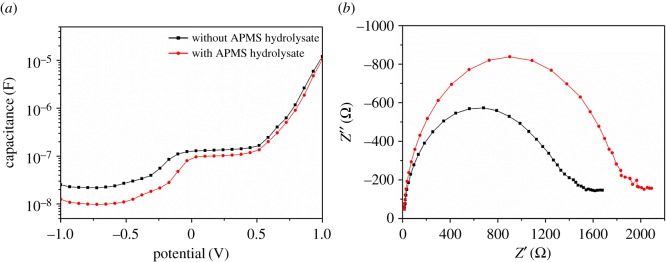
(*a*) Capacitance–voltage characteristics of the PSCs employing TiO_2_ and TiO_2_/APMS hydrolysate as ETLs. (*b*) Nyquist plots of PSCs with bare TiO_2_ (black line) and APMS-hydrolysate-treated TiO_2_ (red line).

The electronic trap states are able to delocalize charge carriers and induce high capacitance at the interface, which can be readily detected using impedance spectroscopy [25]. To further investigate the trap states on the compact TiO_2_ surface both before and after modification, capacitance–voltage measurements were performed on the PSCs fabricated with and without APMS hydrolysate treatment at 1 kHz (figure 5*a*). At this frequency, the capacitance clearly varies with increasing bias voltage, which is indicative of charge accumulation at the compact layer and can thus reflect its capacitance [26]. As shown in figure 5*a*, the capacitances of devices treated with APMS hydrolysate are lower, confirming the passivation effect of APMS hydrolysate. The change in capacitance supports the speculation that fewer carriers gather in the traps and that a longer lifetime results in higher performing PSCs fabricated with APMS-hydrolysate-treated compact TiO_2_.

To further investigate the effect of the APMS hydrolysate at the TiO_2_/CH_3_NH_3_PbI_3_ interface on the photovoltaic performance, electrochemical impedance spectroscopy (EIS) was employed to characterize the charge-transfer dynamics of PSCs by analysing the variation in the impedance related to the different device interfaces. Nyquist plots of the solar cells both with and without the APMS-hydrolysate-treated compact TiO_2_ surfaces under 1 sun illumination are shown in figure 5*b*. Two semicircles are revealed, one in the high-frequency range and another in the low-frequency range, which were measured from 0.1 Hz to 1 MHz. In such PSCs, the resistance at the TiO_2_/CH_3_NH_3_PbI_3_/HTM interface can be determined from the high-frequency (10–100 kHz) semicircle in the Nyquist plots [18,27]. As reflected by the Nyquist plots, the recombination resistance is higher after APMS hydrolysate is grafted onto the TiO_2_ layer, which indicates higher current losses via recombination and an increase in the FF. This increased resistance indicates the retardation of electron back-flow from the TiO_2_ layer into the perovskite [28–30].

## 4. Conclusion

Modifying the interface between TiO_2_ and perovskite layers by inserting APMS hydrolysate was demonstrated to enhance the photovoltaic performance of solution-processed PHJ PSCs. The PCE improved from 13.45 to 15.79% (representing the best solar cells), and the average PCE increased from 12.01 to 14.20%, thus confirming the desired effect of APMS hydrolysate. First, the wettability of GBL on the TiO_2_ surface was enhanced, and as a result, the CH_3_NH_3_PbI_3_ precursor solution spread out over the compact TiO_2_ layer. Second, perovskite crystals were larger and more uniform on the modified layer. The surface traps of TiO_2_ could be passivated by the APMS hydrolysate, and a portion of the molecule was believed to have incorporated into the perovskite crystal. Third, EIS tests revealed that the recombination resistance of the TiO_2_/perovskite interface increased. Our work highlights the effects of APMS hydrolysate on the performance of the TiO_2_/perovskite heterojunction in PHJ PSCs.

## Supplementary Material

Cross-sectional SEM image; J-V curves with 100 millisecond scan delay times

## Data accessibility

The datasets supporting this article have been uploaded as the electronic supplementary material. Our data have been deposited in the Dryad Digital Repository: https://doi.org/10.5061/dryad.8s544 [31].
