# Supplementary material for: Enhancing the efficiency of planar heterojunction perovskite solar cells via interfacial engineering with 3-aminopropyl trimethoxy silane hydrolysate
Source: R Soc Open Sci. 2017 Dec 20;4(12):170980. doi: 10.1098/rsos.170980 (PMC5750005; doi:10.1098/rsos.170980)
Supplement: Cross-sectional SEM image; J-V curves with 100 millisecond scan delay times [file rsos170980supp1.docx]

**Electronic Supplementary Information**

**Enhancing the efficiency of planar heterojunction perovskite solar cells via interfacial engineering with 3-aminopropyl trimethoxy silane hydrolysate**

Ya-Qiong Wang^1,2^, Shou-Bin Xu^2^, Jian-Guo Deng^2*^ and Li-Zhen Gao^1*^

*1 College of Environmental Science and Engineering, Taiyuan University of Technology, Taiyuan, Shanxi, 030024, PR China.*

*2 Institute of Chemical Materials, China Academy of Engineering Physics, Mianyang 621900, Sichuan, PR China*.

[Corresponding author*]

Li-Zhen Gao ([gaolizhen@tyut.edu.cn](mailto:gaolizhen@tyut.edu.cn)).

Fig. S1 shows cross-sectional SEM image of the fabricated cell by inserting PCBM as modified layer. No obvious PCBM modified layer can be observed as Fig. 1(b).


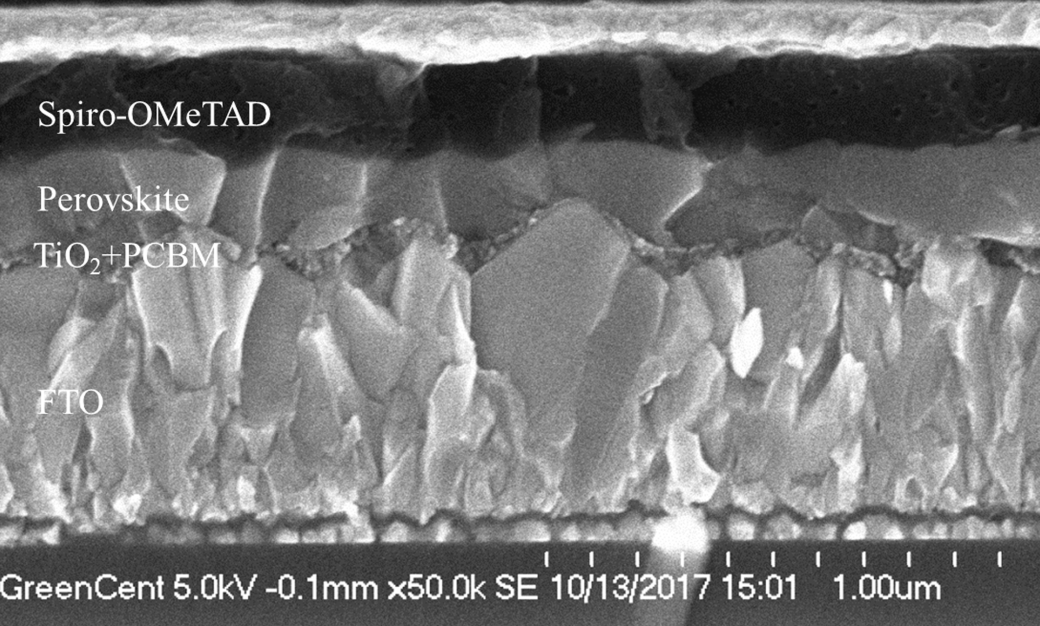


Fig. S1 Cross-sectional SEM image of the fabricated cell by inserting PCBM as modified layer.

Fig. S2 shows the J-V curves with 100 millisecond scan delay times of the best perovskite solar cells fabricated with (a) bare TiO_2_, (b) PCBM-treated TiO_2_ and (c) APMS-hydrolysate-treated TiO_2_, measured under forward and reverse scan. Comparison of the curves under (d) forward scan and (e) reverse scan. It is evident that APMS-hydrolysate is better modified material compared with PCBM as seen in Fig. S2 (d) and (e).

(b)

(a)

(d)

(c)

(e)

Fig. S2 J-V curves with 100 millisecond scan delay times of the best perovskite solar cells fabricated with (a) bare TiO_2_, (b) PCBM-treated TiO_2_ and (c) APMS-hydrolysate-treated TiO_2_, measured under forward and reverse scan. Comparison of the curves under (d) forward scan and (e) reverse scan.
